# Recent Advances in the Catalytic Depolymerization of Lignin towards Phenolic Chemicals: A Review

**DOI:** 10.1002/cssc.202001213

**Published:** 2020-08-03

**Authors:** Xudong Liu, Florent P. Bouxin, Jiajun Fan, Vitaliy L. Budarin, Changwei Hu, James H. Clark

**Affiliations:** ^1^ Key Laboratory of Green Chemistry and Technology Ministry of Education Department of Chemistry Sichuan University Wangjiang Road Chengdu 610064 P.R. China; ^2^ Green Chemistry Center of Excellence Department of Chemistry University of York Heslington York YO10 5DD UK

**Keywords:** Lignin, phenolic monomers, reductive catalytic fractionation, stabilization, valorization

## Abstract

The efficient valorization of lignin could dictate the success of the 2^nd^ generation biorefinery. Lignin, accounting for on average a third of the lignocellulosic biomass, is the most promising candidate for sustainable production of value‐added phenolics. However, the structural alteration induced during lignin isolation is often depleting its potential for value‐added chemicals. Recently, catalytic reductive depolymerization of lignin has appeared to be a promising and effective method for its valorization to obtain phenolic monomers. The present study systematically summarizes the far‐reaching and state‐of‐the‐art lignin valorization strategies during different stages, including conventional catalytic depolymerization of technical lignin, emerging reductive catalytic fractionation of protolignin, stabilization strategies to inhibit the undesired condensation reactions, and further catalytic upgrading of lignin‐derived monomers. Finally, the potential challenges for the future researches on the efficient valorization of lignin and possible solutions are proposed.

## Graphical abstract

**“Lignin‐first” biorefinery**: Catalytic valorization of lignin provides a promising and attractive strategy for producing value‐added phenolic monomers. The recent advances in catalytic depolymerization of technical lignin and reductive catalytic fractionation of protolignin are comprehensively summarized. The mechanism of condensation reactions and possible solutions are also discussed.

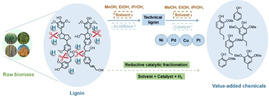

## 1 Introduction

In order to keep up with the growing demand for chemicals and fuels, our alarming reliance on the fossil resources (coal, petroleum and natural gas) need to be mitigated through the development of strategies and technologies enabling the efficient valorization of renewable resources.^1^ The nonedible and attractive lignocellulosic biomass, serves as an ideal feedstock candidate for replacing the fossil resources that deteriorate the environment and aggravate the global warming issues.^2^ 120–130 billion tons of biomass (e. g., corn stover, bagasse and wood chips) were generated each year by photosynthesis in forestry, agriculture and industry domain, whereas only 1 % of the total energy capacity has been efficiently utilized.^3^ In the late 20^th^ century, a traditional refinery protocol has flourished based on the conversion of fossil fuel to bulk chemicals (ethylene, toluene, xylene, etc.), whereas aroused a variety of social issues regarding economic feasibility and environmental sustainability.^4^ Therefore, the development of an integrated biorefinery strategy remains essentially urgent. The transformation of biomass feedstock and its waste stream (black liquor, sawdust) to value‐added chemicals could contribute to alleviating environment concerns and resource scarcity.^5^ Recently, remarkable advances have been achieved towards the biorefinery concept for the utilization of lignocellulosic biomass to produce a wide range of bulk chemicals.^6^ Lignin, as the by‐product of paper mill, is generally burned to generate heat, which is not environmentally benign or sustainable.^7^ Therefore, the exploitation and development of cost‐effective and environmentally sustainable lignin‐first biorefinery strategies for the efficient valorization of lignin to phenolic chemicals and its further upgrading becomes the core and focus among scientific researchers.6b, 8 Emerging lignin valorization strategies are currently revisiting the biorefinery concept.6b From ultimate waste to the most valuable component, the perception of the lignin has evolved over the last two decades. This could be credited to a better chemical structure understanding of the native and technical lignins, but also to the forecasted depletion of aromatic building blocks recently induced by the shale gas revolution.^9^

Though embodying the most abundant renewable aromatic resource in nature, the complexity and heterogeneity of lignin has provoked significant technical challenges for its efficient valorization.^10^ In order to address the problems that lignin causes for the biomass recalcitrance, tremendous efforts and endeavor of the lignin utilization have been invested in the past few decades, including hydrogenolysis,^11^ oxidation,^12^ photocatalytic,^13^ pyrolytic^14^ methods that focused on the efficient depolymerization of lignin to obtain phenolic building blocks.

**Scheme 1:**
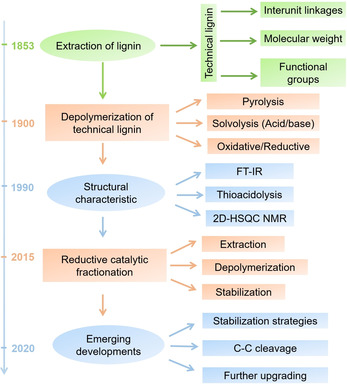
Overview for the chronological development of lignin valorization.

This review attempts to provide a comprehensive overview of the recent research on the valorization of lignin for producing phenolic chemical and bio‐oil, and is structured into three main (interconnected) sections (Scheme 1). Conventional approach‐catalytic depolymerization of technical lignin (Section 3), emerging approach‐reductive catalytic fractionation (Section 4), stabilization strategies and further upgrading of main phenolic products (Section 5). Preceding the three main sections (Sections 3–5), a brief introduction on the structural characteristics of native lignin (protolignin) and technical lignin is included (Section 2), which forms the foundation of modern fractionation and depolymerization technologies.

## 2 Structural Characteristics of Various Lignin

### 2.1 Protolignin

The structural characterization of protolignin played a critical role in our perception for potential valorization. Thanks to the development of 2D NMR and the former breakthrough in lignin characterization brought by the thioacidolysis, the structural complexity of lignin became more rationalized and the highly branched statistical representation of native lignin was replaced by more linear, comprehensive structure.^15^ The better understanding of native lignin has certainly contributed to the development of new biorefinery strategies such as the lignin‐first or reductive catalytic fractionation.

According to Ralph and Ragauskas,^16^ lignins structure from lignocellulosic biomass could be divided into three main kinds: hardwood (angiosperm/dicot) lignin, softwood (gymnosperm) lignin and herbaceous (monocot) lignin. Lignin content is generally highest for softwoods (25–31 wt %), followed by hardwoods (16–24 wt %) and herbaceous crops (16–21 wt %).10b, 17 Besides, the relative content of the monolignols (*p*‐coumaryl alcohol/H, coniferyl alcohol/G and sinapyl alcohol/S) vary greatly among the three biomass kinds.^18^ Softwood lignin is mainly composed of G units (>95 %), while hardwood lignin is made up of both G and S units. However, herbaceous crops contain H, G and S units.2b, 7b, 10b, 19

**Figure 1:**
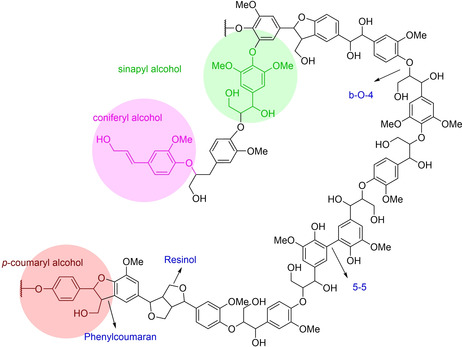
Representative lignin fragment with different phenolic moieties and linkages.

**Figure 2:**
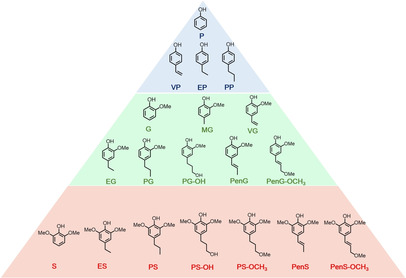
Chemical structures of all the phenolic monomers mentioned in this Review.

Therefore, structural differences were prominent due to the varieties of the monolignols and interunit linkages. Different phenolic units were semi‐randomly cross‐linked by CC_α_−C_β_OC_α_−C_β_C bonds and C−C bonds (Figure 1).^20^ The most abundant β‐O‐4 ether bonds with comparatively lower bond dissociation energy (BDE) play a decisive role in influencing the yield of phenolic monomers.^21^ The yield of phenolic monomers and bio‐oil is highly dependent on the characteristics of the biomass feedstock.^22^ Bouxin et al. reported that the yield and selectivity of alkylphenols during catalytic depolymerization of various lignins depended on the abundance of β‐*O*‐4 linkages.21d Hardwood lignin (50–65 wt %) possess higher content of β‐O‐4 linkage than softwood lignin (43–50 %) due to the highest percentage of S‐type unit lignin of the hardwood, eliminating the possibility of 5–5 or β‐5 radical coupling during its biosynthesis (Table 1).2c On the other hand, the greater number of recalcitrant C−C linkages with higher BDE values (5‐5, β‐β, β‐5, β‐1) are more likely to appear during the biosynthesis of softwoods G‐type unit‐rich lignin.6b, 23 Thus it is generally acknowledged that catalytic hydrogenolysis of lignin in hardwood (poplar, birch, eucalyptus, oak) produced a higher yield of phenolic monomers than that from softwood (spruce and pine). The chemical structures of all the phenolic monomers discussed in this review are listed in Figure 2.

**Table 1:** Distribution of monolignols and interunit linkages in softwood, hardwood, and grass lignin (cited from Refs [6b,19]).

Component	Type		Percentage of total amounts [%]			Bond dissociation
			Softwood	hardwood	grass	energy [kcal mol^−1^]
monolignol	H (*p*‐coumaryl alcohol)		<5	0–8	5–33	–
	G (coniferyl alcohol)		>95	25–50	33–80	–
	S (sinapyl alcohol)		0	46–75	20–54	–
linkages	C−O−C	β‐O‐4	43–50	50–65	74–84	56.54–72.30
		α‐O‐4^[a]^	5–7	<1	n.d.	48.45–57.28
		4‐O–5	4	6‐7	n.d.	77.74–82.54
	C−C	5–5	5–7	<1	n.d.	114.9–118.4
		β–β	2–6	3–12	1–7	–
		β–5	9–12	3–11	5–11	125.2–127.6
		β–1	1–9	1–7	n.d.	64.7–165.8
	others		16	7–8	n.d.	‐

[a] Only present in the dibenzodioxocin moieties (5‐5+α‐O‐4+β‐O‐4)

### 2.2 Technical lignins

Technical lignins can be classified into two main kinds according to the fractionation methods. One is extracted lignin (soda, Kraft, sulfite lignin/lignosulfonate, organosolv lignin, ionic liquid lignin, deep eutectic solvent (DES)‐extracted lignin) while the other lignin remained in residues after the removal of carbohydrates via hydrolysis (hydrolytic lignin).10b, 21b, 24 The pulp industry (Kraft, soda, bisulfite) is the largest producer of technical lignins with Kraft pulping dominating the market.

In 1853, soda pulping was introduced at industrial‐scale on non‐woody lignocellulosic biomass, such as straw, bagasse and flax.^25^ During the soda pulping process, the feedstock are mixed with sodium hydroxide at 160 °C or lower.^26^ The lignin dissolution in alkaline conditions was promoted by the cleavage of β‐*O*‐4 ether linkages, allowing the ionization of free phenolic groups.^27^ Under alkaline conditions, the cleavage of lignin‐carbohydrates complexes (LCC), the depolymerization of lignin and undesired condensation reactions took place simultaneously.^28^ As illustrated in Table 2, Zhao et al. reported severe structural modifications of the spruce and eucalyptus lignins during alkali extraction process, induced by the depletion of β‐O‐4 linkages and the appearance of aryl enol ether moieties.^29^

**Table 2:** Structural characteristics of various technical lignins as function of biomass feedstock and type of pretreatment.

Biomass	Pretreatment	Native linkages (per 100 Ar)^[a]^			Process‐induced linkages (per 100 Ar)			Molecular weight		Functional groups			Ref.
feedstock	process	β‐O‐4	β‐β (β‐β′)^[b]^	β‐5	atilbene	aryl enol ether	phenyl‐ glycerol	*M*_W_ [g mol^−1^]	IP^[c]^	COOH	aliph‐OH	Ph‐OH	
softwood	Kraft	3.2	2.4 (3.2)	0.8	4.8	1.3		6000	6.2	0.5	2.6	2.1	[32]
spruce	Kraft	n.d^[d]^	6.2	3.1		6.2	1.5						[29]
eucalyptus	Kraft	n.d	12.9	n.d									
spruce	Alkali	6	6.3	5.2		13.2	8.4						
eucalyptus	Alkali	n.d	12.7	n.d.			8.9						
													
softwood	Kraft (Indulin AT)	6.1	1	0.3	2.3			4290	8.1	0.33	1.79	2.77	[24b]
mixed straw	Alkali (Protobind)	3.4	0.7	0	0			3270	5.2	0.8	1.26	2.86	
mixed straw	Alcell	5.3	2.8	0.8	0.4			2580	4.3	0.22	1.04	3.3	
wheat straw	organosolv	4.3	0.1	4.5	0.4			1960	4.4	0.21	1.27	2.54	
poplar	organosolv	0.1	1.1	1.8	0			2180	3.8	0.07	0.8	2.59	
spruce	organosolv	0	0.2	3.3	0.7			2030	4.9	0.06	1.43	2.73	
switchgrass	ionic liquid	48	3	10				1362	2.4				[35]
eucalyptus	ionic liquid	57	9	2				844	1.9

[a] Per 100 aromatic rings. [b] Hydrolyzed form of the resinol (β‐β) moiety. [c] Index of polydispersity. [d] not detected.

In the early 1890s, Kraft pulping technology became the most widely used pulping process. During the Kraft process, the raw biomass undergoes harsh treatment in the presence of sodium hydroxide and sodium hydrosulfide mixture (white liquor) at 170 °C, introducing sulfur on the side‐chain of phenolic moieties and leading to similar side reactions than in soda pulping.^30^ Besides, the recovery and separation of Kraft lignin from the black liquor is not so widespread due to a dearth of economic viability and environmental sustainability.^31^ Crestini et al. analyzed the softwood Kraft lignin using quantitative NMR and reported severe structural alterations as only little amount of β‐O‐4, β‐β and β‐5 linkages were detected in the technical lignin.^32^

In 1930, sulfite lignin/lignosulfonate became the historically predominant available technical lignin. During the sulfite pulping process, wood lignin was cooked at 140–170 °C in an aqueous solution of a sulfite or bisulfite salt of sodium, magnesium or ammonium.^33^ Unlike the Kraft lignin, the sulfonic groups were incorporated in the aliphatic side‐chain of phenolic moieties, leading to water‐soluble lignosulfonate salt. Despite being a small share of the whole paper industry, the sulfite pulping is currently providing 90 % of the commercial lignin.^34^

In the late 20^th^ century, organosolv lignin appeared to be a promising strategy, which was generally obtained via the selective extraction of lignin in methanol,^36^ ethanol,^37^ isopropanol,^38^ *n*‐butanol,^39^ THF,^40^ or H_2_O/organic co‐solvents,^41^ followed by precipitation, thus a lignin‐rich precipitate was obtained. In 1989, organosolv lignin, was produced in a semi‐commercial scale plant using ethanol‐water Alcell pulping technology.^42^ Structural analysis of the Alcell organosolv lignin showed that the amount of β‐O‐4 linkages were lower than typical native lignin and similar to the Kraft Indulin lignin (Table 2).24b Finally, hydrolytic lignin is generated via the acidic and enzymatic hydrolysis of carbohydrates, thus remaining lignin in the solid residue.

Ionic liquid (IL) has also been applied for the extraction of lignin owing to its eco‐friendly and facile recyclable properties.^43^ Besides, the anions and cations could be responsible for breaking down the recalcitrant plant cell wall, selectively solubilization of lignin, and further disassembly of fractionated lignin fragments.^44^ For example, an increased phenolic OH and reduced aliphatic OH was observed in IL‐pretreated poplar alkaline lignin by Sun and co‐workers, and the significant decrease of molecular weight was ascribed to the cleavage of β‐O‐4 and modifications of β‐β and β‐5 linkages. Investigation on the structural changes of lignin during IL pretreatment process was achieved by in‐situ NMR and pyrolysis‐GC/MS techniques.^45^ Extensive lignin extraction (up to 82 wt %) of eucalyptus wood was reported by Ovejero‐Perez et al. using protic IL.^46^ It was also observed that β‐O‐4, β‐β and β‐5 linkages were severely depleted and more condensed lignin structures were produced due to the acidity of the protic IL. In another study, lignocellulosic biomass were pretreated with cholinium lysinate in mild conditions and the extracted lignin showed low structural alteration compared to the EMAL lignin (Table 2).^35^ The type of IL (protic, aprotic, acid, base) as well as severity of the pretreatment could explain the different observations made on lignin structural alterations.

Deep eutectic solvents (DES), as a promising alternative for ionic liquid, has garnered much attention recently.^47^ Guo et al. achieved the efficient removal of xylan (80.8 %) and lignin (63.4 %) of corncob in benzyltrimethylammonium chloride (BTMAC)/lactic acid (LA) and benzyltriethylammonium chloride (BTEAC)/lactic acid (LA) DES systems, while retaining 94.1–96.9 % of cellulose in the pretreated corncob.^48^ In this work, DES could be recycled and reused five times without the loss of pretreatment performance. Shen et al. applied DES pretreatment to remove lignin and hemicellulose, thereby distinctly reducing “biomass recalcitrance”. DES‐extracted lignin showed well‐preserved structures (i. e., β‐O‐4, β‐β linkages) without contaminated carbohydrates and owned a relatively low and homogeneous molecular weight.^49^ Moreover, the excellent biodegradability and biocompatibility as well as negligible volatility and facile fabrication of DES makes it an environmentally‐friendly and economic‐viable solvent for the efficient fractionation of biomass.^50^

To conclude, the diversity of technical lignins due to the biomass intrinsic variability and extraction processes created a first layer of complexity that restrained the valorization of the lignin. In absence of clear structural analysis, the heterogeneity of the technical lignins made it difficult to rationalize the potential for added value applications such as the production of phenolic monomers. Upgrading strategies were often too lignin specific, limiting the comparison between different approaches. In‐depth structural analysis of technical lignins using 2D NMR and gel permeation chromatography has recently permitted to overcome those issues. As a consequence, a new thrust in lignin valorization has been focused on catalytic depolymerization of technical lignins to value‐added phenolic monomers.

## 3 Conventional Approach – Catalytic Depolymerization of Technical Lignin

The annual output of Kraft lignin is approximately 45 million tons worldwide with the concurrent production of 100–130 million tons of wood pulp owing to the proven and well‐established pulping technology.^51^ Technical lignin (Kraft and soda) was the waste in the paper mill and generally used for burning to generate heat, which was a low‐value utilization method polluting the environment.^52^ Therefore, establishing a value‐added and economic‐viable strategy than directly burning would be extremely urgent. Before the 21^st^ century, a considerable number of researches focused on the catalytic valorization of technical lignins to achieve as high yield of phenolic monomers as possible. As part of the conventional approaches, the pyrolysis, solvolysis and catalytic oxidative depolymerization of technical lignin will be briefly discussed before introducing the most promising reductive catalytic fractionation strategy.

### 3.1 Pyrolysis

The pyrolysis of the lignocellulosic biomass produces gas (bio‐gas), liquid (bio‐oil) and solid residue (bio‐char). However, due to the higher temperature (350–800 °C) as well as the complex nature of the feedstock, the product varieties and distribution could be extremely difficult to control. Besides, the required temperature of pyrolysis is generally higher than the solvolysis temperature, which is uneconomic in terms of energy consumption.^58^ Direct pyrolysis of raw biomass could both generate lignin‐derived phenols and sugar‐derived products simultaneously with low selectivity, thereby posing significant obstacles for downstream separation.^59^ Herein, several examples of pyrolyzing technical lignin was outlined (Table 3).

**Table 3:** Summary of the (catalytic) pyrolysis of technical lignins.

Biomass feedstock	Lignin type	Reactor type	*T* [°C]	Catalyst	Yield [wt %]	Major products^[a]^	Ref.
corncob residue	Organosolv lignin	fixed bed reactor	350	–	16.2 (monomers)	VP, VG	[53]
softwood	alkali lignin	quartz reactor‐GC/MS	650	Al‐MCM‐41	54 (bio‐oil)	Aromatic hydrocarbons	[54]
softwood	alkali lignin	vertical pyrolysis furnace	600	–	5.0 (monomers)	phenols, catechols	[55]
corncob	Hydrolysis lignin			–	20.5 (monomers)	phenols, catechols	
**–**	soda lignin	Py‐GC/MS	800	Y‐zeolite, Mordenite, ZSM‐5	Y‐zeolite>Mordenite> ZSM‐5	phenol, methyl phenol, dimethyl phenol	[56]
corn straw	alkali lignin	fixed bed reactor	450	Mo/TiO_2_	29 (bio‐oil)	VP, VG	[57]

[a] VP: 4‐vinyl phenol; VG: 4‐vinyl guaiacol.

Hu and co‐workers investigated the pyrolysis behavior of organosolv lignin isolated from corncob residue, demonstrating that bio‐oil was abundant in low molecular weight oligomers (200–500 Da) at mild temperature (150 °C) while phenolic monomers was observed at higher pyrolysis temperature (350 °C).^53^ Custodis et al. prepared zeolites (Al‐MCM‐41, Al‐SBA‐15 and Al‐MSU‐J) with different porosity and acidity, among which Al‐MCM‐41 with a high textural porosity/external surface provided the highest bio‐oil yield of 54 wt %.^54^ The product detected covered phenols, alkoxy phenols, ketones, aromatic hydrocarbon and alkoxy aromatics, which constitute the complex components of bio‐oil. Wu and co‐workers achieved 5 wt % and 20.5 wt % yield of phenolic monomers from alkali lignin (softwood) and hydrolysis corncob lignin, which mainly contains phenols, catechols, aldehydes and ketones.^55^ Kumar et al. compared the catalytic fast pyrolysis of soda lignin with three different zeolites (Y‐zeolite, mordenite, ZSM‐5) and Y‐zeolite showed the highest amount of aromatic monomers (phenols, catechols, guaiacols, benzene, etc.).^56^ Dong et al. showed that Mo‐doped TiO_2_ catalyst could significantly increase the yield (26.72 mg per g lignin) and selectivity (89 %) of phenols, especially for monophenols and guaiacols.^57^

These works achieved a moderate yield of phenolic monomers or bio‐oil, whereas the complexity of products could undesirably increase separation difficulty and economic cost for further utilization. Therefore, more attention has been focused on the catalytic solvolysis of lignin to improve the yield of monomers/bio‐oil at relatively mild conditions.

### 3.2 Solvolysis

**Figure 3:**
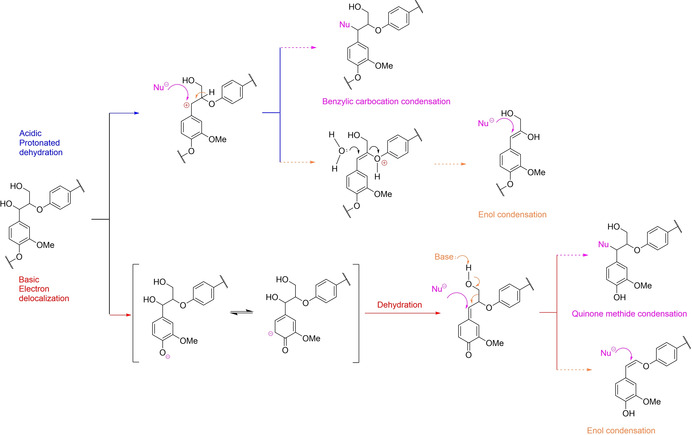
Condensation mechanisms of lignin in acidic and basic conditions.

Lignin solvolysis has been studied for decades with the main interest to produce value‐added chemicals but also for analytical purposes. Lignin acidolysis was one of the first techniques that allows the characterization of lignin.^60^ Hibbert's ketones, discovered in the early 40’s by Harold Hibbert are keto‐containing phenolics monomers generated by the acidolysis of lignin.^61^ Mineral bases were also successfully applied to break down alkyl aryl ether linkages in the so‐called base‐catalyzed depolymerization of technical lignin.^62^ However, the core issue for the efficient solvolysis of lignin lies in stabilizing the intermediates and maximizing the yield of phenolic monomers, which has garnered increasing attention recently. The benzylic position is the source of condensation during lignin extraction (Figure 3). In acidic condition, the carbocation is favorably generated thanks to the delocalization of the positive charge on the aromatic ring.12b, 63 In basic condition, quinone methide is generated via delocalization of the negative charge through the aromatic ring up to the elimination of the benzylic hydroxyl. In both conditions, electrophilic benzylic carbon is then attacked by nucleophiles to form the condensed C−C or C−O bonds.^64^

In the desire to minimize lignin condensation, recent works have been focused on non‐catalytic solvolysis of lignin in the presence of formic acid, which has shown to both catalyze the depolymerization and supply hydrogen to stabilize the reactive products and intermediates.^65^ Other work also highlighted the important role of carbon monoxide generated from formic acid decomposition during non‐catalytic solvolysis of technical lignins.^66^ In the past decade, catalytic oxidative or reductive pathways have been prioritized over non‐catalytic solvolysis for the depolymerization of the lignin. Both strategies have been developed in order to promote lignin depolymerization sometimes through C−C cleavage (oxidation cleavage of C_α_−C_β_) and increase the phenolics yields through reductive stabilization of the intermediates and products.

### 3.3 Catalytic oxidative depolymerization

Historically, the oxidative depolymerization of technical lignin has been focused on the production of vanillin which is considered a key‐intermediate for the manufacturing of bio‐based polymers.^67^ Since 1968, the Borregaard company has been commercially producing vanillin from lignosulfonate in alkali condition using Cu^II^ catalyst in the presence of oxygen.^68^ Other transition metal ions such as Fe^III^, Mn^II, III^, Co^II^ and Zr^IV^ have also been reported to enhance oxygen reactivity and facilitate the cleavage of β‐O‐4 and pinacol C−C linkages of the technical lignin.^69^ More recently, the use of metal oxides (CuO, MnO_2,_ TiO_2_, ZnO) showed that heterogenous catalysts could be as efficient as homogenous metal ion catalysts and facilitate the catalyst recovery.^70^ Other catalysts such as polyoxometalates (POM),^71^ biomimetic catalysts (metallosalen, metalloporphyrins)^72^ have been reported to be able to cleave the β‐O‐4 linkages. However, C−C linkages cleavages using biomimetic catalysts or POM have not been observed or discussed so far.72b Compared to reductive approaches, the oxidative pathway generally requires milder conditions (e. g., reaction temperature around 100 °C), thereby reducing the energy cost.^73^ The other advantage of the oxidative pathway is the production of more valuable aromatic monomers with active functional group (e. g., aldehyde), offering functionalization opportunities.24c On the other hand, limiting the over‐oxidation of the lignin such as ring opening especially when using hydrogen peroxide as oxidant reagent is challenging.^74^ Finally, radical repolymerization of the lignin fragments is also a severe drawback of the oxidative pathway.^75^ Detailed review on the oxidative valorization of lignin have been recently published.70c, 72b, 73, 75, 76

### 3.4 Catalytic reductive depolymerization

Ni, Ru, Pd‐based catalysts were applied to the reductive catalytic depolymerization of organosolv lignin (Table 4). At first, the lignin solubilization is an essential step to achieve high monomers yields. In pure water, Zhang et al. achieved only 6.8 wt % yield of monomers at a low pressure of H_2_ (10 bar) from organosolv birch lignin over Ni_85_Ru_15_with hydrogenated coniferyl and sinapyl alcohol being the major products.^77^ Strüven and Meier conducted the catalytic depolymerization of organosolv beech lignin in H_2_O and 10.1 wt % yield of phenolic monomers was achieved.^78^ In both studies, the limitations of phenolic monomers yield in the above‐mentioned work could be ascribed to the low solubilization of lignin in water.^85^ Besides, they also showed that the presence of hydrogen significantly reduced the formation of coke/tar, which accordingly increased the oil yield.

**Table 4:** Summary of catalytic depolymerization of technical lignin involving hydrogen.

Biomass feedstock	Lignin type	Solvent	Catalyst/ additive	*T* [°C]	t [h]	H_2_ [bar]	Monomer Yield [wt %]	Major products^[a]^	Ref.
birch	organosolv lignin	H_2_O	Ni_85_Ru_15_	130	12	10	6.8	PG‐OH, PS‐OH	[77]
beech	organosolv lignin	H_2_O	Raney Ni	360	3	70	10.1	P, PP	[78]
birch	organosolv lignin	MeOH	Ni_1_−Fe_1_/AC	200	6	20	20.3	PG, PS	[36b]
oak	organosolv lignin	MeOH	Pd/C	180	2	30	25.0	PG, PS	[36c]
corncob	organosolv lignin	MeOH	ZnMoO_4_/MCM‐41	220	4	30	14.3	methyl coumarate, methyl ferulate	[79]
poplar	ammonia lignin	MeOH/H_2_O (1 : 1 v/v)	Pt/Al_2_O_3_	300	2	30	18.9	S, PS, PenS, PS‐OH, PS‐OCH_3_	[80]
oil palm EFB^[b]^	organosolv lignin	EtOH/H_2_O (65 % v/v)	Ru/H_β_	225	6	40	16.5	PG, PenG	[81]
pubescens	organosolv lignin	H_2_O/EtOH (6 : 4 v/v)	Pd/NbOPO_4_	100	20	20	22.4	EP, S	[82]
beech	organosolv lignin	EtOH	sulfided NiMo/ γ‐Al_2_O_3_	300	3	26	4.3	PG, PS	[37b]
bagasse	organosolv lignin	*i*PrOH	Ni/ZrP	260	4	20	15.1	EP	[38]
bagasse	organosolv lignin	*i*PrOH	Ni/MgO	270	4	30	15.0	EP	[83]
corncob residue	organosolv lignin	H_2_O/*n*‐BuOH (4 : 6 v/v)	Ni/HZSM‐5	300	4	20	19.5	EP, EG	[39]
bagasse	organosolv lignin	MIBK^[c]^	H‐USY	350	1	20	19.4	P, G, EP	[84]

[a] P: phenol; EP: 4‐ethyl phenol; PP: 4‐propyl phenol; EG: 4‐ethyl guaiacol; PG: 4‐propyl guaiacol; PenG: 4‐propenyl guaiacol; PG‐OH: 4‐*n*‐propanol guaiacol; S: syringol; PS: 4‐propyl syringol; PS‐OH: 4‐*n*‐propanol syringol; PS‐OCH_3_: 4‐(3‐methoxypropyl)syringol. [b] EFB: empty fruit bunch. [c] MIBK: methyl isobutyl ketone.

In order to enable the primordial solubilization of the lignin before catalytic depolymerization, the subsequent researches on OL catalytic depolymerization were conducted in organic solvents (MeOH, EtOH, *i*PrOH, *n*‐BuOH) or organic/H_2_O co‐solvents.^86^ Zhai et al. prepared different molar ratios of Ni/Fe supported on activated carbon (AC) and Ni_1_Fe_1_/AC gave the highest yield (20.3 wt %) of phenolic monomers with a hydrogen pressure of 20 bar,36b which is in agreement with the work conducted by Sels, proposing that the excessive hydrogen could hamper the hydrogenolysis reaction of lignin due to its negative order property as a function of hydrogen pressure.^87^ Wang et al. achieved 3.9 wt % and 14.3 wt % yield of monomers with N_2_ and H_2_ applied, respectively, which further highlighted the prominent role of H_2_.^79^ Jackson and co‐workers investigated the catalytic depolymerization of ammonia lignin under H_2_ and He atmosphere.^80^ They demonstrated that the total yield of monomers showed an increase trend to reach the highest at 18.9 % with 30 bar of H_2_.

The catalytic conversion of organosolv beech lignin over sulfided NiMo/γ‐Al_2_O_3_ could only afford 4.3 wt % yield of monomers, while direct conversion of beech wood could provide 18.1 wt % yield of monomers under the identical conditions.37b The yield difference between the direct conversion of biomass and a two‐step process involving organosolv isolation and subsequent depolymerization was also observed by Zhai et al. and Wang et al.36b, 79 This suggested that catalytic depolymerization of technical organosolv lignin is more challenging due to severe and irreversible repolymerization reactions during the fractionation of lignin from raw biomass.24b, 36b, 79, 88

Li's group investigated catalytic depolymerization of organosolv bagasse lignin (OBL) in isopropanol. Ni supported on acidic ZrP and alkaline MgO afforded 15.1 wt % and 15.0 wt % yield of phenolic monomers, respectively.^38^, ^83^ In their work, the yield of monomers reached the highest value with a hydrogen pressure of 20 bar for Ni/ZrP catalyst and 30 bar for Ni/MgO catalysts. The yield of biochar continuously decreased upon increasing hydrogen pressure, which could be ascribed to the suppression of condensation reactions induced by unstable lignin‐derived oligomers under the reductive atmosphere. OBL was also subjected to depolymerization in methyl isobutyl ketone (MIBK) and 19.4 wt % yield of monomers was obtained.^84^ In this work, the yield of char also decreased with increasing H_2_ pressure and the total monomer yield obtained with H_2_ nearly doubled than that obtained without H_2_, which highlighted the essential role of hydrogen for the hydrogenolysis of lignin.

The filtrate obtained after the extraction step was directly used as reactant for further depolymerization in some work. Ouyang et al. added H_3_PO_4_ during delignification step and direct catalytic depolymerization of lignin‐rich filtrate over Pd/C gave 25.0 wt % yield of monomers.36c Liu et al. achieved 87.1 % of delignification from corncob residue in H_2_O/*n*‐BuOH co‐solvent and further depolymerization of lignin‐rich filtrate generated 19.5 wt % yield of monomers.^39^ Fang et al. conducted formic acid‐assisted extraction of lignin in H_2_O/EtOH system and filtrate was subjected to catalytic hydrogenolysis, providing 22.4 wt % yield of phenolic monomers at facile condition (100 °C).^82^

The aforementioned work successfully achieved the catalytic hydrogenolysis of organosolv lignin to obtain a moderate yield (3–25 wt %) of monomers involving molecular hydrogen. The metals (Ni, Pd, Pt, Ru, etc.) dissociate molecular H_2_ and the physicochemical properties (acid sites, surface area) of supports (activated carbon, metal oxides, zeolites) contribute to the cleavage of ether bonds. The external hydrogen not only promoted the catalytic hydrogenolysis of lignin, but also inhibited condensation reactions via the hydrogenation of reactive intermediates, thereby reducing the yield of char/coke. Unlike the oxidative approach, the reduction of the side chain functional groups made the product less valuable and could inhibit further functionalization. The main monomers in most work were 4‐alkylguaiacol and 4‐alkylsyringol with ethyl or propyl para‐substituted side‐chain. Several works reported that slightly excessive hydrogen could suppress the catalytic hydrogenolysis of lignin, therefore exploring the optimal hydrogen pressure which both promoted the yield of monomers/bio‐oil and minimized the char formation seems reasonably essential.^38^, ^83^, ^84^

These work reported the catalytic depolymerization of lignin using exogenous hydrogen, which is a fire‐hazard and expensive to handle and transport.^89^ Besides, the production of H_2_ from fossil resources could be expensive and unsustainable.^90^ The catalytic hydrogenolysis of lignin in hydrogen‐donor solvents (methanol, ethanol, isopropanol) without external hydrogen garnered more attention (Table 5).^66^, ^91^

**Table 5:** Summary of catalytic depolymerization of technical lignin without external hydrogen.

Biomass feedstock	Lignin type	Solvent	Catalyst/ additive	*T* [°C]	*t* [h]	Gas/pressure	Monomer yield [wt %]	Major products^[a]^	Ref.
candlenut	organosolv lignin	MeOH	Cu_20_La_20_PMO	310	1	–	34	4‐propyl‐2,3,5 methyl‐phenol	[92]
hardwood	Kraft lignin	H_2_O/MeOH (3 : 1 v/v)	CuMo/ZSM‐5	220	7	Ar	20.6	phenol, 3‐methoxy, 2,5,6‐trimethyl (PMT)	[93]
bamboo	cellulolytic enzyme lignin	MeOH/H_2_O (5 : 2 v/v)	Raney Ni+H‐USY	270	0.5	1 atm N_2_	27.9	EG, PG	[94]
hardwood	Kraft lignin	MeOH	HZSM‐5	220	7	Ar	4.2	G, S	[95]
wheat straw	alkali lignin	EtOH	CuMgAlOx	300	8	10 bar N_2_	23	G, MG	[96]
switchgrass	ionic liquid lignin	*i*PrOH	5 % Ru/C	300	3	20 bar N_2_	27	PenG, PenS, EG	[97]
sorghum	DES extracted lignin	*i*PrOH	5 wt % Ru/C	270	1	N_2_	27.39	P, EP, EG, PG	[47a]
–	Kraft lignin	*i*PrOH/H_2_O	Rh/La_2_O_3_/CeO_2_−ZrO_2_+Fe	373	2	–	26.4	–	[98]
–	lignosulfonate	H_2_O/*n*‐BuOH/*i*PrOH (1 : 1 : 3)	Raney Ni	200	2	1.0 MPa N_2_	11.6	EG	[99]
corn stalk	cellulolytic enzyme lignin	iPrOH/H_2_O (2 : 1 v/v)	Ni_50_Pd_50_/ SBA‐15	220	8	0.5 MPa N_2_	8.14	EP, ES, PS	[100]
birch	acid‐extracted lignin			245	8		18.52	PS	
beech	organosolv lignin	*i*PrOH	Ni/Al_2_O_3_	170	12	10 bar N_2_	13.4	PenS, sinapyl alcohol, coniferyl alcohol	[89]

[a] P: phenol; EP: 4‐ethyl phenol; G: guaiacol; MG: 4‐methyl guaiacol; EG: 4‐ethyl guaiacol; PG: 4‐propyl guaiacol; PenG: 4‐propenyl guaiacol; S: syringol; ES: 4‐ethyl syringol; PS: 4‐propyl syringol; PenS: 4‐propenyl syringol.

Warner et al. applied CuLa‐doped PMO catalyst for depolymerization of organosolv candlenut lignin and demonstrated that in‐situ hydrogen from methanol reforming effectively suppress condensation reactions.^92^ Ekhe and co‐workers performed catalytic depolymerization of Kraft lignin in H_2_O/MeOH co‐solvents without addition of hydrogen and obtained high yields of alkylphenols with minimum char formation.^93^ Methanol reforming and condensation mechanism of lignin was proposed in the same group.^95^ The combination of Raney Ni and H‐USY outperformed Raney Ni or H‐USY alone for the catalytic hydrogenolysis of lignin due to synergistic catalytic effect.^94^ During catalytic process, Raney Ni acted as lignin‐cracking and methanol‐reforming catalyst and H‐USY as acidic catalyst for the cracking of ether bonds. Cu‐ and Ni‐based catalysts undertake the responsibility of promoting methanol‐reforming processes to produce in situ hydrogen, which promote the hydrogenolysis of ether bonds in lignin.^101^

Huang et al. reported that one‐pot conversion of soda lignin in supercritical ethanol (sc‐EtOH) over CuMgAlOx resulted in high monomers yield (23 wt %) without char formation.^96^ The minimized char formation could be associated with that the in‐situ produced hydrogen suppressed the condensation reactions of unstable intermediates. Ethanol plays a direct role in the solvolysis of lignin and indirectly serve as hydrogen‐donating solvent for hydrogenolysis of lignin and products stabilization.

Isopropanol is widely acknowledged for the catalytic transfer hydrogenolysis (CTH) of lignin.91c, 102 CTH of lignin was employed to valorize the lignin‐enriched residues from the ionic liquid (IL) pretreatment by Kim et al.^97^ The higher amount of hydrogen produced with Ru/C promoted the hydrogenolysis of lignin. Das et al. achieved 27.4 wt % yield of phenolic compounds over Ru/C from DES‐extracted sorghum lignin.47a The composition analysis of gas products confirmed that hydrogen released from catalytic dehydrogenation of *i*PrOH promoted the hydrogenolysis. Jin and co‐workers performed catalytic depolymerization of Kraft lignin in the mixture of H_2_O and *i*PrOH and the availability of H_2_ could be controlled by changing the ratio of H_2_O/*i*PrOH.^98^ Several other works also corroborated the in‐situ hydrogen released from isopropanol, which further promoted the hydrogenolysis of lignin.^89^, ^99^, ^100^

To conclude, all of the above work substantiated that in‐situ hydrogen from the reforming of alcohol at facile conditions facilitated the hydrogenolysis of lignin, which unlocked the hydrogen‐free depolymerization of lignin methodology. Nevertheless, several studies pointed out that the condensed structure of the technical lignin (e. g. low amount of β‐O‐4 linkages) significantly restrained the yields of monomers. Considering the limitation of the conventional approach, the following section will introduce the emerging lignin‐first strategy. Other strategies such as C−C linkages cleavages could also bring a solution and will be later discussed.

## 4 Emerging Strategy – Reductive Catalytic Fractionation

### 4.1 Introduction of “lignin‐first” strategy

The reductive catalytic fractionation (RCF) takes its route from the organosolv process (Alcell). The previous strategy was to fractionate the biomass components (e. g., organosolv) prior to catalytic depolymerization in order to reduce the complexity of downstream separation.^103^ The purpose of the organosolv (Alcell) process was to improve the enzymatic saccharification of high value cellulose and the technical lignin was sold as low‐grade fuel.^104^ This reductive catalytic fractionation approach completely changes this dogma, in which the lignin is now considered as the high value biomass component. In 2015, the group of Sels and Abu‐Omar proposed the “reductive catalytic fractionation” strategy based on the “lignin‐first” biorefinery principle (Table 6).^105^ The lignin could be dissolved and depolymerized in the form of phenolic monomers, dimers and oligomers in a reductive atmosphere with the catalysts, while retained the carbohydrates in the solid residues. The reducing agent could be the molecular hydrogen, the alcohol solvents (MeOH, EtOH, *i*PrOH, etc.) and an internal hydrogen‐donor derived from the biomass (formic acid). The extraction of lignin from lignocellulosic biomass, the subsequent catalytic hydrogenolysis of lignin‐derived oligomers and further stabilization of phenolic monomers are involved during the process.22b, 106 In this section, the heterogenous catalysis was exclusively discussed due to its preferential use for reductive depolymerization of lignin. Nevertheless, several works utilizing homogenous catalysts (e. g., Ru/Ir complex, B(C_6_F_5_)_3_) under redox‐neutral conditions have also been recently reported but achieved relatively lower yield of phenolic monomers (generally below 10 wt %).^107^

**Table 6:** Summary of reductive catalytic fractionation of lignin involving hydrogen.

Biomass feedstock	Solvent	Catalyst/additive	*T* [°C]	*t* [h]	H_2_ [bar]	Monomer yield [wt %]	Major products^[a]^	Carbohydrate retention [wt %]	Ref.
birch	MeOH	Ru/C	250	3	30	51	PG, PS	94 (C_6_)+63 (C_5_)^[b]^	[105a]
poplar	MeOH	ZnPd/C	225	12	34	54	PG, PS	carbohydrates 74	[105b]
poplar	MeOH	Pd/C+ H_3_PO_4_/NaOH	200	3	20	48	PG‐OH, PS‐OH	cellulose 98 hemicelluloses 90	[108]
eucalyptus	MeOH	Ni@ZIF‐8	260	8	30	44.3	PG, PS, PG‐OH, PS‐OH	cellulose 90 hemicelluloses 67	[109]
birch	MeOH	Pd/C+Yb^III^–triflate	180	2	30	55	PS, PS‐OH, PS‐OCH_3_	glucan∼100 xylan 97	[21c]
black locust	MeOH	Pd/C	250	2	20	35.1 (oil)	PG, PS	–	[110]
corn stover	MeOH	Ni/C+H_3_PO_4_	200	6	30	38	methyl coumarate, methyl ferulate	cellulose 44 hemicellulose 1	[111]
birch	MeOH	Ru/C	250	3	30	48	PG, PS	93 (C_6_)+69 (C_5_)	[112]
		Pd/C				49	PG−OH, PS−OH	94 (C_6_)+81 (C_5_)	
birch	MeOH	Ni/Al_2_O_3_	250	3	30	44	PG−OH, PS−OH	glucan 93 xylan 83	[113]
apple	MeOH	Mo_*x*_C/CNT	250	3	10	42	PenG, PenS	98 (C_6_)+89 (C_5_)	[114a]
eucalyptus	MeOH	MoO_*x*_/SBA‐15	260	4	30	43.4	PenG−OCH_3_, PenS−OCH_3_	98 (C_6_)+89 (C_5_)	[114b]
eucalyptus	BuOH/H_2_O (1 : 1 v/v)	Ru/C	200	2	30	48.4	PG−OH, PS−OH	cellulose 96 hemicelluloses 15	[87]
birch	MeOH/H_2_O (7 : 3 v/v)	Pd/C+H_3_PO_4_	200 (*T*_1_) 180 (*T*_2_)	3	30	37	PS, PS−OH, PS−OCH_3_	glucan 92	[115]
poplar	MeOH	Ni/C	190	3	30	17.2	PG−OH, PS−OH	–	[116]

[a] PG: 4‐propyl guaiacol; PS: 4‐propyl syringol; PG‐OH: 4‐propanol guaiacol; PS‐OH: 4‐propanol syringol, PS‐OCH_3_: 4‐(3‐methoxypropyl)syringol; PenG: 4‐propenyl guaiacol; PenS: 4‐propenyl syingol; PenG‐OCH_3_: 4‐(3‐methoxypropenyl)guaiacol; PenS‐OCH_3_: 4‐(3‐methoxypropenyl)syringol. [b] C_6_: Glucose, galactose; C_5_: xylose, arabinose.

In 2015, Van den Bosch et al. achieved 51 wt % yield of phenolic monomers and 14 wt % yield of dimers with 98 wt % of delignification degree in methanol.105a Parsell et al. explored the synergistic effect of Pd/C and ZnCl_2_ on the cleavage of β‐O‐4 linkages, finding that 54 wt % yield of phenolic products was provided with nearly 100 % selectivity towards 4‐propyl guaiacol (PG) and 4‐propyl syringol (PS).105b The effects of H_3_PO_4_ or NaOH on the delignification and yield of phenolic monomers has been investigated by Renders et al., and observed that both acidic and basic additives could enhance the delignification. The distinct difference is that H_3_PO_4_ results in a higher yield of phenolic monomers in oil compared with neutral condition, but NaOH leads to a significant loss of cellulose and promotes the repolymerization thus produced lower yield.^108^ Liu et al. performed the catalytic depolymerization of lignin in eucalyptus sawdust using Ni@ZIF‐8 catalyst and obtained 44.3 wt % yield of phenolic monomers with 95 wt % delignification degree.^109^ The 4‐propyl guaiacol and 4‐propyl syringol occupied 55 % among the phenolic monomers. In 2017, the group of Hensen found that the metal triflates combined with Pd/C could give a 55 wt % yield of aromatic monomers. During the disassembly process of lignin, metal triflates are more active for the cracking of β‐O‐4 ether bond than Pd/C while Pd/C is mainly responsible for cleaving α‐O‐4, 4‐O‐5 and β–β linkages.21c 35.1 wt % yield of bio‐oil was obtained from black locust bark compared with that from black locust wood and the presence of suberin and a more condensed lignin structure in bark was highlighted.^110^ Anderson et al. investigated the addition of an acid co‐catalyst (H_3_PO_4_ or acidified carbon support) on the yield of phenolic monomers and found that the yield increased from 27.2 wt % to 38 wt % with H_3_PO_4_ added.^111^ The acid co‐catalyst was observed to be effective for promoting lignin solvolysis and accelerating the cleavage of ester bonds of coumarate and ferulate structure in corn stover. However, the acid also promoted the significant dissolution of hemicelluloses and cellulose, which is not beneficial for the selective conversion of lignin.

Sels and co‐workers observed that the total yield (nearly 50 wt %) of monomers were quite similar for Ru/C and Pd/C. Regarding the distribution of monomers, Ru/C showed 75 % selectivity towards 4‐propyl phenolics (PG/PS) due to the efficient hydrogenolysis of C_γ_‐OH, while Pd/C favors the formation of propanol‐substituted phenolics (PG‐OH, PS‐OH) with 91 % selectivity.^112^ In 2017, the same group developed a catalyst pellet with the catalyst fixed in a reactor basket to ease the catalyst recycling and a clean catalyst‐free pulp was obtained, which addressed the downstream separation issues during RCF.^113^ In 2018, they explored the fractionation of lignin from eucalyptus in H_2_O/BuOH co‐solvent, which achieved 96.9 wt % of delignification degree with concurrent 85.2 wt % conversion of hemicellulose.^87^ The less carbohydrates (especially C_5_ sugars) remained in the pulp induced by the addition of H_2_O will be discussed in detail in Section 4.4. The above work utilizing lignin‐first strategy achieved an appreciable yield of saturated side‐chain phenolic monomers, however, Mo species (Mo_*x*_C or MoO_*x*_) were reported to maintain side‐chain C=C bonds and even promote the etherification to produce monolignols ethers.^114^

Except for the liquid‐phase batch reactors, flow‐through reaction systems have been studied recently which addressed the difficulties for the stabilization of lignin intermediates and the separation of catalysts from biomass feedstock. Kumaniaev et al. employed a transfer hydrogenolysis step in a flow‐through system to achieve a 37 wt % yield of monomers.^115^ Ni/C catalyst could give a cumulative yield of 17.2 wt % monomers with the high catalyst loading in a flow‐through reactor.^116^ Though overwhelming advantages the flow‐through system shows, the yield of phenolic monomers is significantly lower than that obtained in batch reactors possibly due to the repolymerization of lignin in the non‐catalytic solvent system without a reductive atmosphere, which resulted in the formation of more recalcitrant C−C bonds.^106^

Some work reported the hydrogen‐free depolymerization of lignin from raw biomass, as shown in Table 7. In 2013, Xu and co‐workers achieved 50 wt % yield of phenolic monomers in MeOH, while 48 wt % and 27 wt % yield of monomers could be obtained in EtOH and *i*PrOH, respectively.^117^ They also demonstrated that alcohols provide sufficient active hydrogen species since the addition of external hydrogen has no effect on lignin conversion, which is further confirmed by isotopic tracing experiments. In 2015, an expanded work exploring the effect of biomass type and catalyst loading was investigated by Abu‐Omar and co‐workers.^118^ In their work, birch (32 wt %) results in higher monomer yields than those found for poplar (26 wt %) and eucalyptus (28 wt %).

**Table 7:** Summary of reductive catalytic fractionation in inert atmosphere.

Biomass feedstock	Solvent	Catalyst/ additive	*T* [°C]	*t* [h]	Gas/pressure	Monomer yield [wt %]	Major products^[a]^	Carbohydrate retention [wt %]	Ref.
birch	MeOH	Ni/C	200	6	1 atm Ar	54	PG, PS	‐	[117]
	EtOH					48	PG, PS		
	*i*PrOH					27	PG, PS, PenG, PenS		
birch	MeOH	Ni/C	200	6	2 bar N_2_	32	PG, PS,	‐	[118]
poplar						26	PenG, PenS		
eucalyptus						28	PenG, PenS		
birch	MeOH/H_2_O (1 : 2 mol/mol)	Pt/γ‐Al_2_O_3_	230	3	30 bar N_2_	49	PG, PS	glucan 41 xylan <1	[91a]
birch	EtOH/water (1 : 1 v/v)	Pd/C	195	1	4 bar Ar	49	PenS	‐	[90]
pine						23	PenG		
s‐birch	EtOH/water (1 : 1 v/v)	Pd/C	210	15	Ar	36 mol %	PS, PenS	glucan 81.5 xylan 2.4	[91b]
f‐birch						35 mol %	PS, PenS	glucan 80.9 xylan 2.1	
poplar						22 mol %	PG, PS	glucan 81.1 xylan 1.0	
spruce						12 mol %	PG	glucan 76.1 xylan 1.0	
pine						7 mol %	PG	glucan 76.4 xylan 1.1	
birch	EtOH/H_2_O (1 : 1 v/v)	Ar	200	4	Co‐phen/C	34	PS, PenS	glucan 18.5 xylan 1.3	[119]

[a] PG: 4‐propyl guaiacol; PS: 4‐propyl syringol; PenG: 4‐propenyl guaiacol; PenS: 4‐propenyl syringol.

Recently, Ouyang et al. achieved 49 % yield of phenolic monomers with 82 % selectivity towards 4‐propyl syringol in the mixture of methanol/H_2_O (1 : 2 v/v) under hydrogen‐free condition.91a They demonstrated that the selectivity towards propenyl and propyl‐substituted monomers could be tuned by adjusting the ratio of MeOH/H_2_O and reaction temperature. To summarize, the catalytic transfer hydrogenolysis of lignin was promoted by in‐situ hydrogen generated from the methanol reforming process.

Galkin and Samec reported that 23 % yield of 4‐propenyl guaiacol and 49 % yield of 4‐propenyl syringol could be obtained from pine and birch wood without external hydrogen in H_2_O/EtOH co‐solvent, respectively.^90^ They illustrated that hydrogen is most likely from formic acid generated during the pulping process could hydrogenolysis the β‐O‐4 bond but not sufficient to hydrogenate the propenyl group. Utilizing a part of the lignocellulose as an internal source of hydrogen for the reductive lignin transformations was substantiated by Galkin et al.91b and Cao et al.^120^ Rautiainen et al. also observed that the addition of formic acid/sodium formate significantly improved monomer yields from 5 wt % to 34 wt % and cobalt catalyst contributed to the depolymerization of lignin fragment via catalytic transfer hydrogenolysis.^119^ This strategy unlocked the novel methodology for the fractionation of lignin and selectively conversion to aromatic monomers.

### 4.2 Alkylation during RCF process

**Figure 4:**
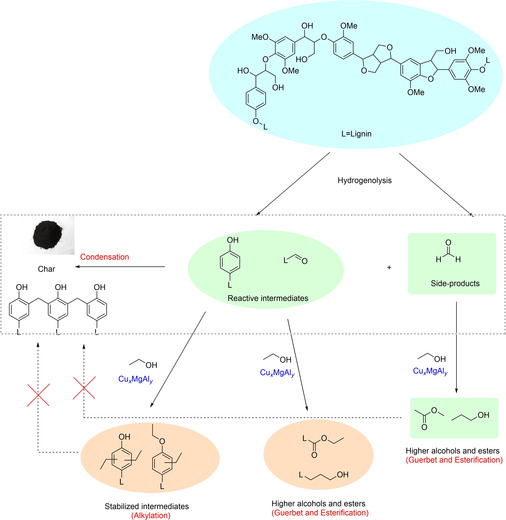
Proposed reaction network for stabilizing the reactive intermediates (adapted from Ref. [121a] with permission, copyright from American Chemical Society, 2015).

Inspired by the principles of alkylation protection protocol, Hensen and co‐workers reported the C‐ and O‐alkylation using ethanol reagent to restrain the condensation reactions (Figure 4).^96^, ^121^ On the top of being one of the most suitable solvent for solvolysis of the lignin, ethanol played three essential roles that contributes to both depolymerization and stabilization processes. First, the reforming of ethanol produced hydrogen which serves as a reducing reagent for the hydrogenolysis/hydrogenation of lignin. Second, ethanol could be responsible for stabilizing the aromatic aldehydes and formaldehyde via aldol condensation reaction, thereby preventing the repolymerization reaction. Third, ethanol served as a capping agent to stabilize the active phenolic intermediates by C‐alkylation of aromatic ring and O‐alkylation of phenolic hydroxyl group.121b

**Figure 5:**
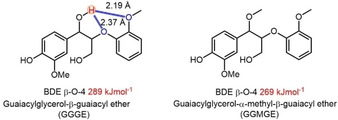
Hydrogen bond lengths and BDEs in non‐methylated and methylated structures (adapted from Ref. [122] with permission, copyright from Wiley, 2016).

Chen et al. reported the conversion of α‐methylated β‐O‐4 model compounds and confirmed the lower bond dissociation energy (BDE) by DFT calculation (Figure 5).^122^ Methylated structure (GGMGE) could give 20 kJ mol^−1^ lower BDE value than GGGE, which could be explained by intramolecular hydrogen bond between the proton of C_α_‐OH and the oxygen at β‐O‐4 (OH_α_−O_β_), the oxygen at aromatic methoxy group (OH_α_−O_methoxy_). The methylated of active Ar‐OH could destroy the intramolecular hydrogen bond, thereby lowering the BDE value of the C_β_−O bond to facilitate the β‐O‐4 cleavage.11a, 123 α‐Methoxylated β‐O‐4 intermediate was observed in the lignin oil by Van den Bosch et al., which was derived from the birch sawdust in pure methanol.^113^ In 2018, a robust and scalable butanosolv pretreatment was established by Westwood and co‐workers, which can be further oxidized to generate functionalized material.12c In 2019, Ragauskas and co‐workers developed acid‐catalyzed diol pretreatment of eucalyptus lignin and 1,4‐butanediol (BDO) pretreated lignin retained higher amount of β‐O‐4 linkages than ethanol pretreated lignin, indicating that 1,4‐BDO quenched the benzyl carbocation species and formed ether linkages with a hydroxyl tail at the C_α_ position of side‐chain.^124^ Recently, the group of Deuss applied four primary alcohols (ethanol, *n*‐propanol, *n*‐butanol, *n*‐pentanol) during organosolv fractionation process and observed etherified structure at benzylic C_α_ position, which could not only protect β‐O‐4 motif but also enhance delignification degree under pretreatment condition.^125^

### 4.3 Typical phenolic monomers produced during RCF

**Figure 6:**
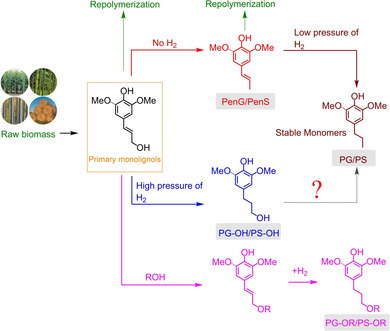
Typical monomers produced during RCF (adapted from Ref. [87] with permission, copyright from Royal Society of Chemistry, 2018).

The phenolic monomers including propenyl‐substituted phenols (PenG, PenS), propyl substituted phenols (PG, PS), the direct hydrogenation products of monolignols/propanol‐substituted phenols (PG‐OH, PS‐OH), saturated and etherified product (PG‐OR, PS‐OR) were generally observed during the RCF process (Tables 6 and 7). Sels and co‐workers discovered the distribution of phenolic monomers could be highly dependent on the gas atmosphere (H_2_ or N_2_) and its pressure.^87^ If no hydrogen was applied, the hydrogenolysis of monolignols to produce propenyl‐substituted phenols was the dominant pathway (Figure 6). If lower pressure of H_2_ (5 bar) was adopted, further hydrogenation of unsaturated C_α_−C_β_ bonds could be observed. However, the direct hydrogenation of monolignols (coniferyl alcohol/sinapyl alcohol) to produce propanol‐substituted phenols predominates the reaction pathway at higher pressure (>10 bar), which could be explained by the difference of H_2_‐reliant properties between hydrogenolysis and hydrogenation reactions.^126^

It has been reported that the extraction and depolymerization of lignin could initially produce primary monolignols (*p*‐coumaryl, coniferyl, sinapyl alcohol) as reactive intermediates. However, the unsaturated side‐chain C=C bonds could undergo radical repolymerization to produce higher molecular weight oligomers, which goes against the lignin‐first biorefinery strategy to obtain value‐added phenolic monomers.22b, 113, 127 Therefore, the selective hydrogenation of side‐chain C=C bonds while maintaining the benzene ring could efficiently suppress the oligomerization reactions.64a

### 4.4 Fate of carbohydrates during RCF

In the desire to valorize the whole lignocellulosic biomass, the fate of hemicellulose and cellulose during RCF has been carefully assessed from previous work.21c, 105, 108, 111 As illustrated in Tables 5 and 6, the retention of cellulose and hemicellulose fluctuated depending on the process conditions. The retention of cellulose maintained more than 90 wt % in pure methanol, whilst showed a slight decline as the ratio of H_2_O increased. In pure methanol, the retention of hemicellulose exhibited a decreasing trend with temperature increasing. Less retention of hemicellulose could be obtained when enhancing the percentage of H_2_O and near‐complete removal of hemicellulose occurred in pure H_2_O, which could be ascribed to the cleavage of ester and ether linkages between lignin and hemicellulose (so‐called lignin‐carbohydrate complexes, LCC).^128^ The significant difference between the retention of cellulose and hemicellulose during RCF could be attributed to the more refractory structure of semicrystalline cellulose, which undesirably hinders its solvation behavior.1c, 88

**Figure 7:**
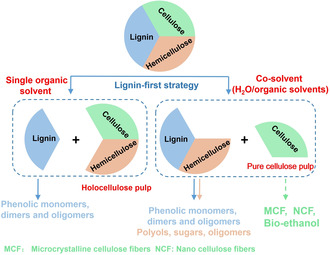
Schematic representation of two “lignin‐first” biorefinery protocols.

Sels and co‐worker investigated the effect of bio‐based solvents on the delignification, yield of phenolic monomers and carbohydrate retention. MeOH and ethylene glycol (EG) outperformed other solvents (H_2_O, EtOH, 2‐PrOH, 1‐BuOH, THF, Diox, Hex) employed in terms of “lignin‐first delignification efficiency” (LFDE), which takes three main factors; degree of delignification, hemicellulose removal and cellulose retention, into consideration.^129^ The carbohydrates are converted to corresponding polyols like pentitols (xylitol, etc.) and hexitols (sorbitol, mannitol, etc.) with a small quantities of C_4_ and C_3_ polyols. The synergistic effect of alcohol/H_2_O mixture on the RCF of poplar was investigated in MeOH/H_2_O and EtOH/H_2_O co‐solvents with different volume ratios by the same group.^88^ The retention of cellulose remained stable irrespective of varying the ratio of MeOH/H_2_O and EtOH/H_2_O, while the hemicellulose content in the pulp could be controlled by altering the percentage of H_2_O. In 2018, Renders et al. achieved 85.2 wt % conversion of hemicellulose in H_2_O/*n*‐BuOH co‐solvent (1 : 1 v/v) with C_5_ polyols obtained, while retaining 96.4 wt % of cellulose in the pulp.^87^ The solvents system provide possibility and potential for us to tailor the composition of the pulp to satisfy downstream application demands (Figure 7).

## 5 Other Strategies for Improving the Monomer Yields and Potential Upgrading of Main Phenolic Products

### 5.1 In situ stabilization of the polymeric lignin

**Figure 8:**
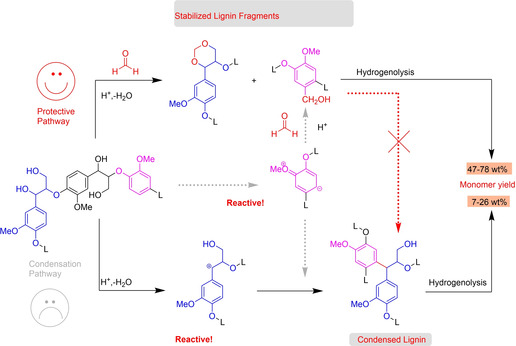
Reaction mechanisms for FA‐stabilized lignin fragments (adapted from Ref. [130] with permission, copyright from American Association for the Advancement of Science, 2016).

The new strategies of reductive fractionation of the lignin proposed to depolymerize and stabilize lignin by mixing metal catalysts with the biomass which often resulted in impossible recovery of catalyst. This severe drawback lead to the development of flow system where the two‐stage lignin extraction and depolymerization provided lower yield due to unavoidable condensation of the lignin during extraction. In 2016, Shuai et al. proposed a novel strategy involving the addition of formaldehyde to inhibit the condensation reactions by forming the 1,3‐dioxane acetal structure during the lignin extraction process (Figure 8).^130^ This was a serious breakthrough to consider the formaldehyde being the perfect protecting reagent than the ideal reactant for the polymerization of lignin.

The acetal formation during extraction prevented the lignin condensation and also avoided the cleavage of the b‐O‐4 linkages, enhancing the lignin potential for monomers production. Catalytic hydrogenolysis of the formaldehyde‐protected lignin produced 3–7 times higher yield (47–78 wt %) of phenolic monomers than in the absence of protection (7–28 wt %).^130^ Afterwards, they compared different protecting reagents (aldehydes, ketones, dimethyl carbonate, phenylboronic acid), with formaldehyde giving the highest yield (46 wt %) of phenolic monomers, followed by propionaldehyde (42 wt %) and acetaldehyde (37 wt %).^131^ The aldehyde‐stabilized lignin could be readily selectively dissolved in an organic solvent and catalytically depolymerized to a near‐theoretical yield of phenolic monomers (40–50 wt % for a typical hardwood).^132^

### 5.2 Pre‐oxidation of the polymeric lignin

**Figure 9:**
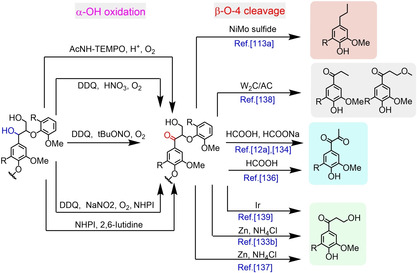
Representative examples of previous methods to depolymerize the benzylic oxidized lignin.

It has been demonstrated that the oxidation of benzylic hydroxyl could both weaken the C_β_−O bonds and suppress the condensation reactions induced by the reactive benzylic carbocations (C_α_^+^).12a, 133 The earliest work on the depolymerization of oxidized lignin was proposed by Stahl group in 2013 and 2014 (Figure 9). High yield of phenolic monomers (52.2 wt %) was achieved from the oxidized aspen lignin at mild temperature (110 °C) in aqueous formic acid solution with α, β‐diketones products occupying 19.8 wt %. This was more than 7 times higher than aromatic monomers achieved from non‐oxidized aspen lignin.^134^ They also have developed several oxidation methods, including stoichiometric oxidation, metal‐catalytic aerobic oxidation and metal‐free catalytic aerobic oxidation.^135^ The same group also investigated the effect of native lignin varieties on the aerobic oxidation‐hydrolysis process, and achieved 42 wt % yield of low‐molecular‐weight aromatics from poplar lignin.^136^ The group of Luterbacher also achieved 36 mol % yield of α, β‐diketones from the DDQ (2,3‐Dichloro‐5,6‐dicyano‐1,4 benzoquinone)‐oxidized lignin (stoichiometric oxidation) and 31 mol % yield from the catalytic oxidized lignin, with 80 % selectivity toward syringyl propane diketone.12a

In 2015, Westwood and co‐workers achieved the chemoselective oxidation of lignin model compounds and native lignin by the DDQ/*t*BuONO/O_2_ system. The extracted lignin was further depolymerized with Zn as catalyst to obtain phenolic monomers with Hibbert's ketones as the major products.133b, 137 Besides, the same group has achieved the selective production of C_α_‐ketones over W_2_C/AC catalyst from beech lignin and the β‐O‐4 model compounds have proved to give higher yield of phenolic monomers.^138^ The Hibbert's ketone products were also obtained by Stephenson's group using Ir catalyst.^139^

High yield of phenolic monomers (32 wt %) could be obtained from birch wood using a two‐step oxidation‐hydrogenation strategy over a NiMo sulfide catalyst by Zhang et al, and it has been proposed the peroxidation of C_α_−OH to C_α_=O could not only lower the BDE values for the C_β_−O bonds but also inhibits the repolymerization due to the inability to generate reactive benzylic carbocations.133a

### 5.3 Stabilization of the monomer through acetal formation

**Figure 10:**
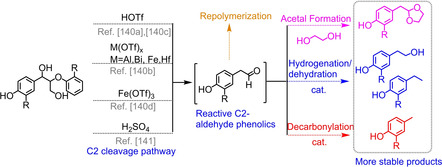
Stabilization pathway of C_2_‐aldehyde intermediates.

In the same acetal protecting strategy, Barta and coworkers have achieved the stabilization of C_2_‐aldehydes with the addition of ethylene glycol (EG) via the acetal formation mechanism (Figure 10), which was the same with the work described by Luterbacher.^140^ Unlike Luterbacher's works, Barta used the acetal formation to stabilize monomeric products instead of polymeric lignin. She employed the acid (HOTf) ‐mediated cleavage of β‐O‐4 and β‐5 model compounds and observed that the C_2_‐aldehydes released from the cracking of β‐O‐4 linkages could be stabilized through the formation of acetal structure with ethylene glycol.140a In order to further substantiate the mechanism, pine, beech, and walnut shell organosolv lignins were used for the acid‐assisted catalytic depolymerization. Three different acetals formed by the aldol condensation between C_2_‐aldehydes and ethylene glycol were produced after the treatment of lignin in ethylene glycol solvent with 7.5 wt % HOTf, which agreed well with the reaction mechanism of the model compounds. The same group also compared the effect of different metal triflates [M(OTf)x] on the yield of C_2_‐aldehydes using β‐O‐4 model compounds, finding that the metal triflates Bi(OTf)_3_, Fe(OTf)_3_, and Hf(OTf)_4_ showed the most promising cleavage efficiency to form C_2_‐aldehdyes with Fe(OTf)_3_ giving the highest yield (19.3 wt %) of phenolic C_2_‐aldehydes.140b Recently, a mild hydrogenolysis method using H_2_SO_4_ was developed with dimethyl carbonate (DMC) as solvent and ethylene glycol as stabilization agent, achieving 77–98 % yield of C_2_‐acetal phenolic monomers.^141^

### 5.4 Cleavage of C−C bonds

To date, most researchers primarily focused on the C−O−C ether bond cleavage in lignin due to its lower bond dissociation energy and higher amount than C−C bond.^142^ However, the design of catalytic systems aiming at further enhancement of monomer yield seems to be more captivating, which could achieve the fullest valorization of lignin. In 2018, Wang and co‐workers achieved the production of vanillin and syringaldehyde from organosolv lignin at room temperature under visible light through oxidative C_α_−C_β_ cleavage.^143^ The photocatalytic aerobic cleavage mechanism was further substantiated by employing β‐1 and β‐O‐4 linked dimer model compounds. Shuai et al. developed an efficient catalytic system showing high conversion of methylene‐linked C−C model dimer to obtain 88 % yield of aromatic monomers over CoS_2_. Further depolymerization of Kraft lignin gave 13.0 wt % yield of aromatic monomers, which doubled more than that obtained with noble metal‐based catalyst (Ru/C).^144^ Recently, Dong et al. reported that up to 32 wt % yield of monocyclic hydrocarbons was achieved over Ru/NbOPO_4_ multifunctional catalyst through the cleavage of both C−O−C and C−C linkage.^145^ The superior catalytic activity of Ru/NbOPO_4_ for the cleavage of C−C bonds was further confirmed using lignin‐derived dimers, which could be ascribed to the strong Brønsted acid sites induced by NbO_*x*_ species and phosphates as well as the activation of hydrogen molecules promoted by Ru nanoparticles.

However, systematic research on the cleavage of C−C interunit linkages in native lignin remains limited and challenging, which could be a hotspot topic in the future research.

### 5.5 Further upgrading to specific chemicals

**Figure 11:**
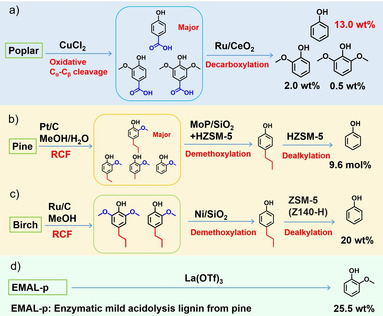
Typical examples for the conversion of lignin and further upgrading to specific chemicals.

Recently, catalytic conversion of lignin in biomass to obtain single compound (phenol, guaiacol, etc.) has received increasing attention. In 2018, 13 wt % yield of phenol was obtained from separated poplar lignin via the combination of C_α_−C_β_ bond oxidative cleavage and subsequent decarboxylation (Figure 11a).^146^ High selectivity towards 4‐propyl guaiacol was obtained through the Pd/C‐catalyzed reductive depolymerization, followed by MoP/SiO_2_‐catalyzed demethoxylation and zeolite‐catalyzed dealkylation (Figure 11b).^147^ Sels and colleagues performed the catalytic upgrading of lignin oil obtained from the reductive catalytic fractionation of birch wood to obtain 20 wt % yield of phenol via demethoxylation and dealkylation (Figure 11c).^148^ These works achieved an impressive yield of bio‐based phenols from raw biomass via (i) RCF combined with demethoxylation/dealkylation; and (ii) oxidative depolymerization followed by decarboxylation. La(OTf)_3_ could catalyze the depolymerization of lignin to obtain alkyl‐guaiacol and alkyl‐syringol, which underwent further dealkylation and demethoxylation to obtain an appreciable yield (25.5 wt %) of guaiacol (Figure 11d).^149^ Ferulic acid could be derived from ferulate structure in herbaceous biomass,16a and Brønsted acid catalyzed defunctionalization of ferulic acid to bio‐catechol was reported, which unlocked the potential to produce catechol from raw biomass.^150^ All of the above work bridge the gap between lignin and bio‐based phenols through funneling and defunctionalization of a mixture of phenolic monomers.

On the other hand, Barta and co‐workers is looking at atom‐economy pathways that permit rapid conversion of dihydroconiferyl alcohol to high value products (e. g., amines) that can enter the chemicals supply chain at much later stage than bulk chemicals (e. g., phenol).2a, 151 The selective transformation of lignin‐derived phenolic monomers to terephthalic acid (TPA) was also developed.^152^ Despite the fact that intensive efforts have been made to further upgrading and funneling of phenolic mixtures to pure phenols, the phenolic monomers were also applied for manufacturing specialized functional materials to keep pace with massive demand for polymer materials and commodities, such as resins, thermoplastics, adhesives, coatings.^153^

## 6 Summary and Outlook

The Review provides an overview of the lignin valorization methods and strategies at different stages pertaining time‐honored to the state‐of‐the‐art. Both catalytic depolymerization of technical lignin and reductive catalytic fractionation (RCF) of protolignin in raw biomass achieved an appreciable yield of phenolic monomers. Generally, RCF of protolignin provided a higher yield of phenolic monomers than the hydrogenolysis of technical lignin under identical conditions, which could be ascribed to the more recalcitrant and condensed structure of technical lignin induced by undesirable structural modifications (e. g., condensation reactions) during the extraction step. Cleavage of C−C bonds could be a promising strategy to overcome the recalcitrance of the technical lignin. The major obstacle of the lignin‐first strategy is the fate of the carbohydrates. Both yield of phenolic monomers and retention of carbohydrates should be equally considered. The chemical stabilization and further upgrading of phenolic monomers should also be highly prioritized. Though great and marvelous advancements have been achieved, challenges for future researches have been identified:

1. The understanding of structural characteristic in protolignin and its interaction with other biopolymers (cellulose and hemicellulose) faces great difficulties due to the occurrence of unavoidable modification and undesired condensation reactions during the sample preparation process (e. g., ball milling). In‐depth comprehension of structural features in protolignin could guide us to a better design of catalytic process for the valorization of lignin. The development of in situ GPC and NMR technology could assist the further understanding of structural variations during the catalytic process.
2. The cost and recovery of metal catalysts during the RCF of lignin are limiting the development of the process. Most studies achieving a high depolymerization degree of lignin utilized noble‐metal (Pd, Pt, Ru) catalysts. Exploitation of cheaper transition‐metal (Ni, Fe, Cu) catalysts for the efficient hydrogenolysis of lignin enhanced the economic viability. The difficulties for the thorough isolation of catalysts from the reaction residues impede catalyst recycling. Though the flow‐through reaction system could address the catalyst separation problems, irreversible and undesired repolymerization reaction are inevitable during the extraction step. While showing promising results to overcome those issues, the strategy to stabilize lignin during extraction and depolymerization involving extra step needs to be economically viable at scale.
3. Even if high selectivity toward phenolic monomers (propyl or propenyl syringol and guaiacol) was achieved from raw biomass, the costly separation from the crude products remains a major drawback. The applications of obtained phenolic monomers as a bulk should be prioritized. The polymer materials that could be manufactured from phenolic moieties are widely used in our daily life, such as phenol–formaldehyde (PF) resin, PET, phenolic‐derived rubber, etc. The exploration for the value‐added application of the phenolic monomers with special functional groups to synthesize high‐value materials could drive the advancement of the “lignin‐first” strategy.

## Conflict of interest

The authors declare no conflict of interest.

## Biographical Information

*Xudong Liu received his bachelor's degree in chemistry from Sichuan University, China, in 2017. He is currently pursuing his PhD under the supervision of Prof. Changwei Hu at the same university. He started to conduct research at the University of York, United Kingdom, as a visiting PhD student under the supervision of Prof. James Clark and Dr. Jiajun Fan since August, 2019. His research interests include the catalytic depolymerization of lignin in raw biomass and hydrogenation/ hydrodeoxygenation of lignin‐derived model compounds*.

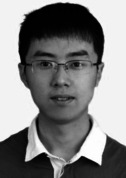

## Biographical Information

*Dr Florent Pierre Bouxin conducted his PhD on lignin valorization at the French National Institute of Agricultural Research (INRA) and the University of Reims, France, under the supervision of Dr Patrice Dole and Prof Jean‐Hugues Renault in 2011. Then, Dr Bouxin acquired extensive experience in the breakdown and fractionation of recalcitrant lignocellulosic biomass through his PDRA position at the Lawrence Berkeley National Lab, USA, under the supervision of Dr Anthe George, working on the production of drop‐in molecules from industrial relevant lignin. He is currently working at the Green Chemistry Centre of Excellence (York, United Kingdom) on the conversion of the lignocellulosic biomass using microwave technology under the supervision of Prof. James H. Clark*.

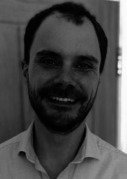

## Biographical Information

*Professor Changwei Hu is a Member of CCS (Chinese Chemical Society), ISC3 (International Sustainable Chemistry Collaborative Center) and Fellow of RSC Member. He is recipient of Excellent National Teacher of Teaching and Special Allowances Expert of the State Council in China. He is now the director of the Key Laboratory of Green Chemistry and Technology (Ministry of Education) at Sichuan University. His research areas include catalytic conversion of bio‐based materials to fuel and useful chemicals, catalytic functionalization of C−H bonds, catalytic activation of small molecules especially greenhouse gases, molecular modeling of catalytic systems, and other green chemistry interrelated researches. He has published more than 340 papers with 6000 citations with 30 patents authorized, above 10 times awarded by MOE (China) and Sichuan Province (China)*.

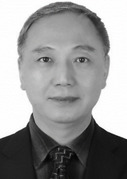

## Biographical Information

*James Clark is a Professor at the University of York and is founding director of the Green Chemistry Centre of Excellence and Bio‐renewables Development Centre. His research involves the application of green chemical technologies to waste or low value feedstocks to create new green and sustainable supply chains for chemical and material products. He has published over 500 articles (H index 76) and written or edited over 20 books. He has received numerous awards including Honorary Doctorates from universities as well as prizes from the RSC, SCI, ACS, and EU. In 2018 he won the RSC Green Chemistry Prize*.

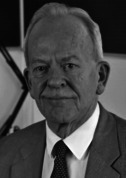
